# Genome-Wide Identification and Transcriptional Analysis of the MYB Gene Family in Pearl Millet (*Pennisetum glaucum*)

**DOI:** 10.3390/ijms24032484

**Published:** 2023-01-27

**Authors:** Miaohong Lin, Zhuoyan Dong, Hongkai Zhou, Guanyu Wu, Liang Xu, Sheng Ying, Miao Chen

**Affiliations:** 1 College of Agricultural Sciences, Guangdong Ocean University, Zhanjiang 524091, China; 2 Noble Research Institute LLC, 2510 Sam Noble Parkway, Ardmore, OK 73401, USA

**Keywords:** pearl millet, PgMYB gene, abiotic stress, transcriptional analysis

## Abstract

The MYB gene family widely exists in the plant kingdom and participates in the regulation of plant development and stress response. Pearl millet (*Pennisetum glaucum (L.)* R. Br.), as one of the most important cereals, is not only considered a good source of protein and nutrients but also has excellent tolerances to various abiotic stresses (e.g., salinity, water deficit, etc.). Although the genome sequence of pearl millet was recently published, bioinformatics and expression pattern analysis of the MYB gene family are limited. Here, we identified 208 PgMYB genes in the pearl millet genome and employed 193 high-confidence candidates for downstream analysis. Phylogenetic and structural analysis classified these PgMYBs into four subgroups. Eighteen pairs of segmental duplications of the PgMYB gene were found using synteny analysis. Collinear analysis revealed pearl millet had the closest evolutionary relationship with foxtail millet. Nucleotide substitution analysis (Ka/Ks) revealed PgMYB genes were under purifying positive selection pressure. Reverse transcription-quantitative PCR analysis of eleven R2R3-type PgMYB genes revealed they were preferentially expressed in shoots and seeds and actively responded to various environment stimuli. Current results provide insightful information regarding the molecular features of the MYB family in pearl millet to support further functional characterizations.

## 1. Introduction

Transcription factors (TFs) are essential proteins in the regulation of gene expression and biological function by binding to cis-regulatory elements in the promoter region of genes. MYB TFs were first identified in viruses [1] and are present in almost all eukaryotes as one of the largest TF families [2]. The structure and function of MYB are conserved in plants compared to animals and yeast. Based on the number of the conserved structural domains, the MYB family can be classified into four subfamilies, such as MYB-related (1R-MYB), MYB-R2R3 (2R-MYB), MYB-R1R2R3 (3R-MYB), and MYB-4R [3]. In plants, the first identified MYB gene is COLORED1 (C1), which participates in anthocyanin biosynthesis in maize kernels [4]. To date, MYB family members have been identified and characterized in more than 75 plant species [5], such as in *Arabidopsis thaliana* [6], *Oryza sativa* [7], *Brachypodium distachyon* [8], and *Setaria italic* [9] (Table S1). Based on extensive computational analysis, MYB-R2R3 is reported to be the largest subfamily of the plant MYB family. Recent evolutionary study suggests the older MYB-R2R3 subfamily is usually associated with development and stress resistance, while the newly formed subfamily is more involved in regulating metabolic processes [5].

MYB genes, especially MYB-R2R3 members, aid plants coping with the globe climate crisis by regulating the expression of stress-related genes. In *Arabidopsis*, several MYB genes have been demonstrated to have critical roles in response to abiotic stresses. For example, *AtMYB14* and *AtMYB15* are involved in cold stress tolerance by negatively regulating the expression of dehydration-responsive element-binding factors (DREB) [10,11]. Overexpression of abiotic stress-responsive *AtMYB* genes can significantly enhance drought tolerance in transgenic *Arabidopsis* [12,13,14,15]. In crops, rice *OsMYB60* and *OsMYB55* and wheat *TaMYB31* function as key regulators, positively improving resilience to water stress [16,17,18]. Notably, a foxtail millet MYB gene, *SiMYB19*, improves salt tolerance in transgenic rice by promoting abscisic acid (ABA) accumulation, thus, upregulating ABA-dependent stress responsive genes [19].

Pearl millet (*Pennisetum glaucum (L.)* R. Br.) belongs to the *Panicoideae* subfamily of the C_4_ grass family (*Poaceae*) with high photosynthetic efficiency; thereby, it can be used as a sustainable alternative energy source. As the sixth most productive cereal crop, pearl millet is widely grown in arid and semi-arid regions, with more than 300 million hectares in major growing regions, such as Africa and Indonesia [20,21]. In addition to its high nutritional value, pearl millet is also rich in micronutrients (e.g., iron and zinc) [22]. It has good environmental adaptability and can survive in harsh environmental conditions, such as low soil fertility, high saline soils, high temperature, and water deficit. More importantly, its genome has been sequenced and annotated, making pearl millet ideal for studying stress tolerance mechanisms in cereal crops [23,24]. Several gene families (i.e., WRKY and NAC) have been characterized in pearl millet [25,26]; however, knowledge of the MYB family and its involvement in abiotic stress response remains limited.

Here, we identified 193 high-confidence pearl millet MYB genes and categorized them into four subgroups based on the protein structural analysis. Synteny analysis revealed 18 pairs of segmentally duplicated PgMYB genes, and pearl millet MYB genes exhibited a tight collinear relationship with that of foxtail millet. Expression analysis of eleven R2R3 type PgMYB genes by reverse transcription-quantitative PCR showed most of them were preferentially expressed in aboveground tissues and dry seeds and actively responded to different environmental stimulus, such as salinity, water limitation, and deleterious temperature. The current findings may contribute to understanding the substantial functions of PgMYB genes in responding to the unprecedented climate changes.

## 2. Results

### 2.1. Genome-Wide Identification and Phylogenetic and Structural Analysis of Pearl Millet MYB Genes

A total of 208 putative MYB candidates were identified from a public database (http://cegsb.icrisat.org/ipmgsc/, accessed on 20 August 2022) [27] (Table S2, Supplementary Files S1–S3). These PgMYB candidates were present on all seven chromosomes, and based on their chromosomal location, they were designated as *PgMYB1*–*PgMYB208* (Figure S1). Note that due to the incompleteness of their genome sequence, 15 PgMYB candidates (e.g., *PgMYB5*, *PgMYB17*, *PgMYB35*, *PgMYB38*, *PgMYB49*, *PgMYB67*, *PgMYB106*, *PgMYB109*, *PgMYB110*, *PgMYB118*, *PgMYB143*, *PgMYB150*, *PgMYB174*, *PgMYB188*, and *PgMYB194*) were excluded from downstream analysis. Then, we utilized the NCBI-CDD (https://www.ncbi.nlm.nih.gov/cdd/, accessed on 20 August 2022) and SMART (http://smart.embl-heidelberg.de/, accessed on 20 August 2022) online programs to identify the MYB signature domain (i.e., MYB_DNA binding structural domain). The results indicated PgMYB candidates were classified into four subgroups according to the numbers of MYB structural domains, including ninety-three MYB-related, ninety-five MYB-R2R3, three MYB-R3, and two atypical MYB genes. Additionally, the average length of R2 and R3 structural domains is 50–55 amino acids (Figure S2), which is similar to that of in other species, suggesting the MYB-R2R3 protein structural domain is evolutionarily conserved.

**Figure 1:**
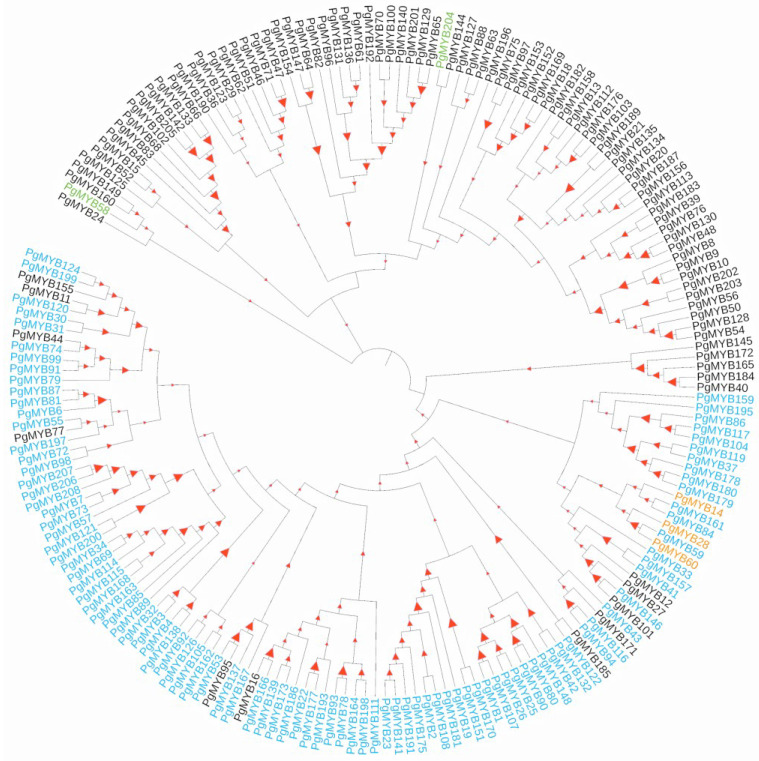
Phylogenetic analysis of PgMYB gene family. Phylogenetic tree was constructed using MEGA11 with 1000 bootstrap replications based on a full-length amino acid sequence alignment of 193 high-fidelity PgMYB proteins. Members belong to MYB-R2R3, MYB-related, and MYB-3R, and atypical subfamilies are represented in blue, black, orange, and green fonts, respectively. The higher the bootstrap value for a particular branch, the larger the size of the red triangles.

**Figure 2:**
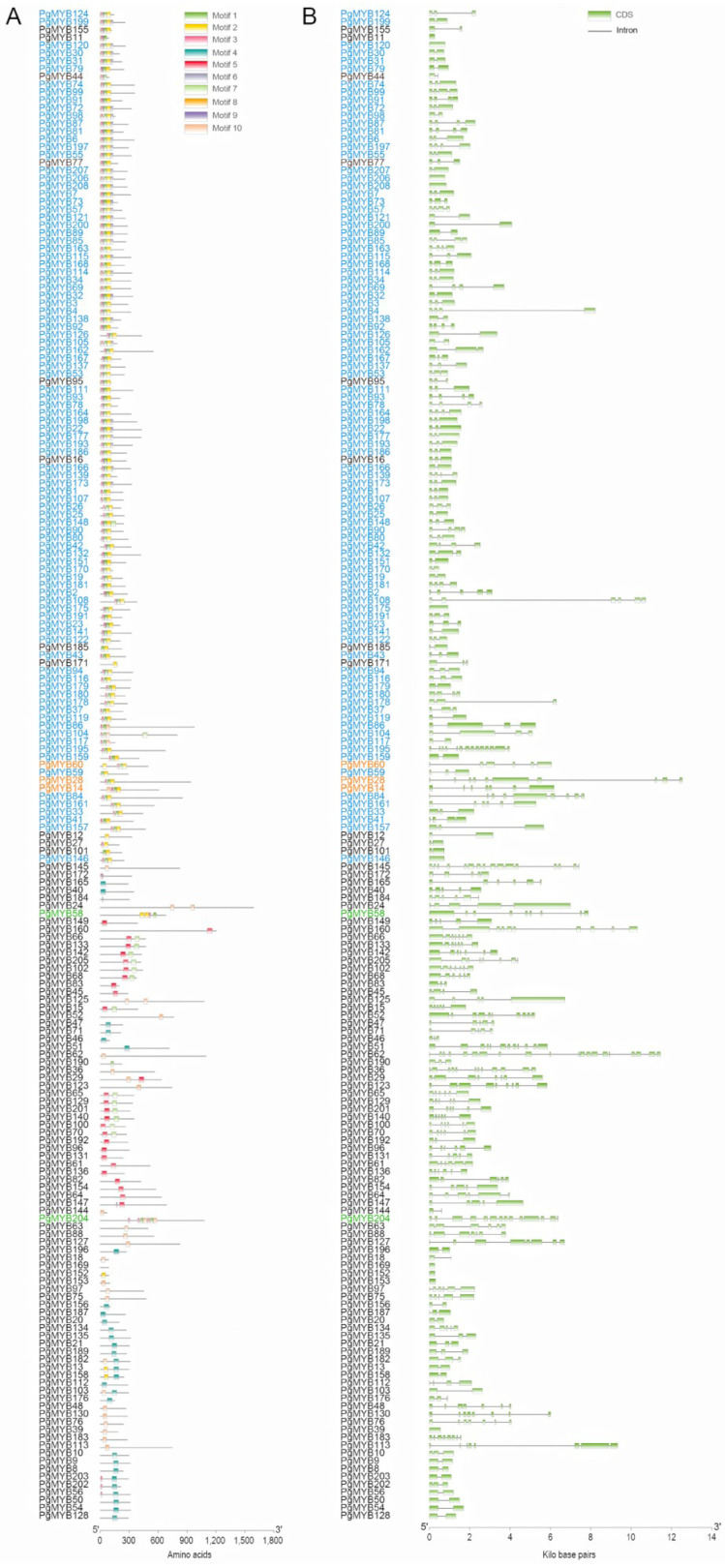
Distribution of conserved motifs and gene structural analysis of PgMYB gene family. (**A**) The conserved motifs of PgMYB proteins were predicted by MEME Suite 5.4.1 and visualized using TBtool. (**B**) Exon–intron distribution of PgMYB genes. Exons and introns are represented by green rectangle boxes and black lines, respectively. Members belong to MYB-R2R3, MYB-related, and MYB-3R, and atypical subfamilies are represented in blue, black, orange, and green fonts, respectively.

A phylogenetic tree was constructed to examine the evolutionary relationship among the 193 high-fidelity PgMYB candidates using full-length protein sequence alignments (Figure 1). The results showed the MYB-related and R2R3 members were clearly grouped into distinct clades, which is consistent with the prediction of motif structural analysis. Ten conserved motifs, which are in a range between eight to fifty amino acids, were identified by MEME Suite 5.4.1. As shown in Figure 2A and Figure S3, none of the motifs ubiquitously appeared in all PgMYB proteins. Motif 3 was the most common motif that appeared in 110 PgMYB proteins, while motif 7 presented only in 15 PgMYB members. In addition, motifs 1, 2, and 3 had the closest associations and tightly presented in 99 PgMYB proteins, while motif 4 and motif 5 were only present in MYB-related subgroup members.

Exon–intron distributions of PgMYB genes were examined to determine their structural diversity. Ten PgMYB genes had no intron, and about 52% (100/193) of PgMYB genes contained two or three exons (Figure 2B, Table S2).

### 2.2. Subcellular Localization Analysis of PgMYB Candidates

We utilized the WoLF PSORT online program (https://wolfpsort.hgc.jp/, accessed on 20 August 2022) to predict the subcellular localization of the PgMYB candidates. The result indicated most PgMYBs (173/193) localized to the nucleus, while eight, nine, and three PgMYBs, respectively, localized to the chloroplast, cytoplasm, and mitochondria (Table S2). Notably, all non-nuclear localized PgMYBs belong to MYB-related subgroups.

### 2.3. Synteny Analysis of the PgMYB Candidates

Gene duplication is the main source mechanism for new genes and plays a vital role in the evolution and expansion of plant gene families [28]. Thus, we utilized the MCScanX program [29] to study the replication events occurring in pearl millet. Eighteen pairs of segmental duplicated PgMYB genes were identified, whereas no tandem duplication was found (Table 1). The substitution rate (non-synonymous/synonymous, Ka/Ks) was used to determine the selective pressure during genome evolution after gene duplication events [30]. Ka/Ks ratios for all eighteen duplicated gene pairs were below 1 (between 0.14 and 0.62), indicating gene functional conservation during evolution and suggesting the pearl millet MYB gene family underwent purifying selection after the duplication events. The divergence time for each pair of PgMYB genes ranged from 27.2 to 163.8 million years ago (MYA).

**Figure 3:**
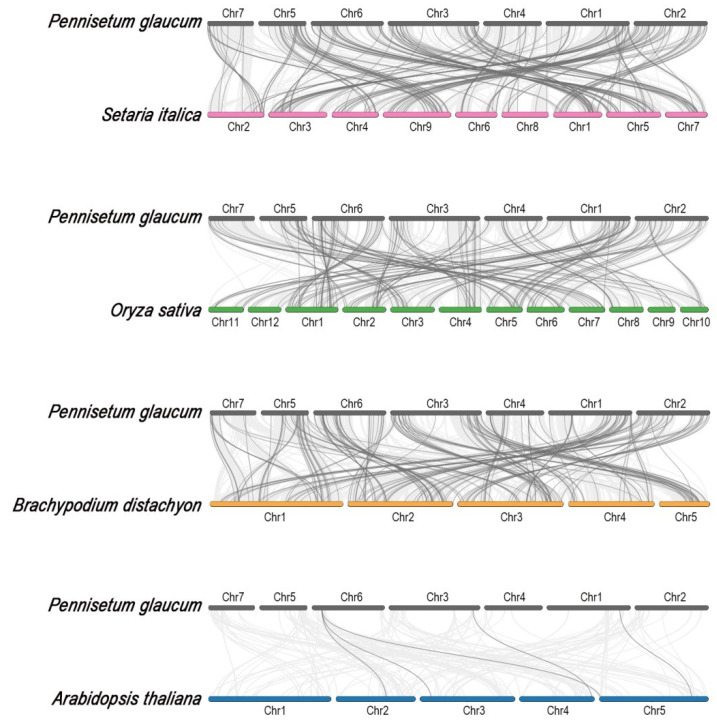
Synteny analysis of MYB genes between pearl millet and other model species. The light gray lines in the background indicate the collinear region within pearl millet and foxtail millet (pink), rice (green), *Brachypodium* (orange), and *Arabidopsis* (blue) genomes, while dark gray lines indicate syntenic MYB gene pairs, respectively.

We next investigated the orthologous relationship of MYB genes between pearl millet and other model species (Figure 3). A total of two hundred one, one hundred ninety, one hundred sixty, and five ortholog MYB gene pairs were respectively identified between pearl millet and foxtail millet (*Setaria italica*), *Brachypodium* (*Brachypodium distachyon*), rice (*Oryza sativa*), and *Arabidopsis* (*Arabidopsis thaliana*). It is not a surprise that pearl millet had the most MYB ortholog pairs with fox millet because of the closest kinship (Tables S3–S6). Ka/Ks analysis of orthologous MYB gene pairs in pearl millet and four other species revealed the rates of synonymous substitution in pearl millet were slightly lower than those observed in foxtail millet and *Brachypodium* (Figure S4).

### 2.4. Transcriptional Analysis of Stress Responsive PgMYB Candidates

**Figure 4:**
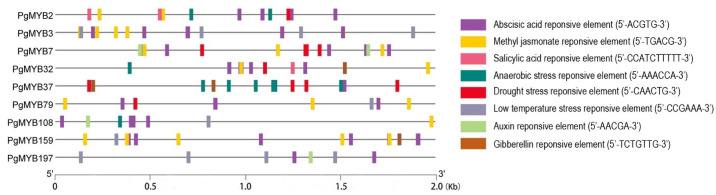
Promoter analysis of nine R2R3-type PgMYB genes. Abiotic stress-related *cis* elements are predicted using PlantCARE program. Conserved sequence of each cis-element is listed in parentheses.

To characterize the PgMYB genes in response to abiotic stresses, we first examined the distribution of stress-related cis-regulatory elements in their promoter regions (2 kb upstream of start codon) (Figure 4, Table S7). At least one abiotic stress-responsive cis-elements, such as methyl jasmonate (MeJA), salicylic acid (SA), drought- and low-temperature-responsive elements, was found in the promoter region of each PgMYB gene, suggesting they might broadly participate in distinct stress response. Notably, abscisic-acid- (ABA) responsive element (ABRE) was the most widespread cis-element, present in 88% (169/193) of PgMYBs’ promoters. MYB-R2R3 subgroup members are known to functionally modulate the plant abiotic stress response. Thus, we decided to examine the expression changes of nine R2R3-type PgMYB candidates (e.g., *PgMYB2*, *PgMYB3*, *PgMYB7*, *PgMYB32*, *PgMYB37*, *PgMYB79*, *PgMYB108*, *PgMYB159*, and *PgMYB197*) whose closest homologs in *Arabidopsis* have been shown to be in response to multiple abiotic stresses (Figure S5) [10,14,31,32,33,34,35].

**Figure 5:**
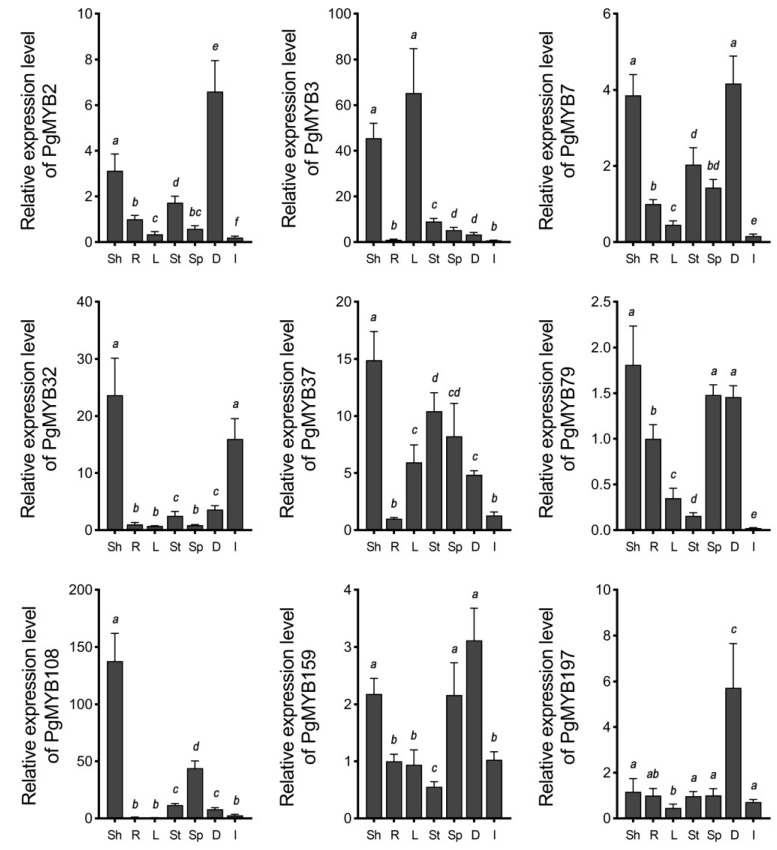
Tissue-specific expression analysis of nine R2R3-type PgMYB genes. Expression level of each PgMYB gene in root is set as control. Sh and R, shoot and root of three-week-old seedling; L, mature leaves; St, stems; Sp, spikes; D, dry seeds; I, geminating seeds. The data represent the mean values of three replicates ± SD. Statistical significance of differences was tested by one-way ANOVA analysis (*p* < 0.05) and is indicated by lower case letters.

We first examined the transcript levels of these PgMYB genes in the shoots and roots of three-week-old seedlings, the leaves, stems, and spikes of mature plants, and dry and germinating seeds using RT–qPCR (Figure 5). The results indicated that except for the *PgMYB197* gene, transcript levels of other genes were higher in shoots than that in roots. Additionally, tissue-specific expression pattern was observed in mature plants. For instance, *PgMYB3* and *PgMYB108* genes were expressed only in leaves and spikes, respectively. It is worth noting the transcript level of the *PgMYB32* gene accumulated during germination, while all remaining PgMYB genes exhibited high abundance in dry seeds.

**Figure 6:**
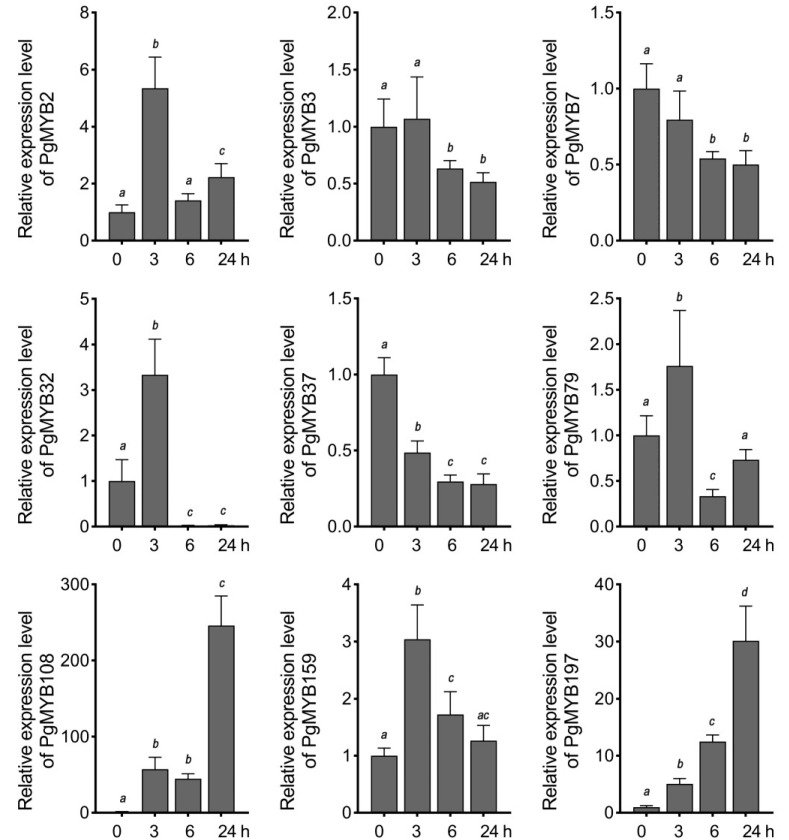
Expression analysis of nine R2R3-type PgMYB genes in response to ABA treatment. Three-week-old hydroponically grown seedlings were treated with 100 µM ABA. Shoot tissues were harvested at each indicated time point. Expression level of each PgMYB gene at 0 h is set as control. The data represent the mean values of three replicates ± SD. Statistical significance of differences was tested by one-way ANOVA analysis (*p* < 0.05) and is indicated by lower case letters.

**Figure 7:**
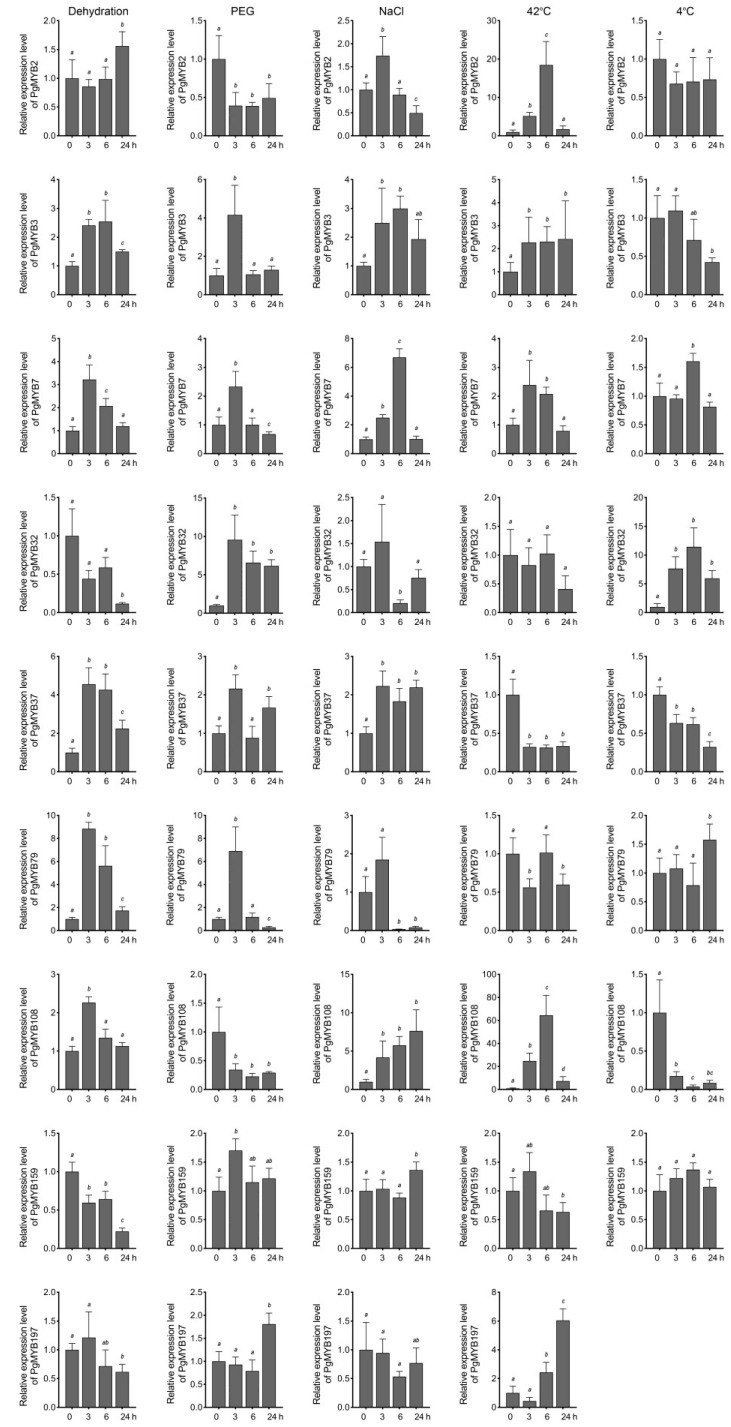
Expression analysis of nine R2R3-type PgMYB genes in response to different abiotic stresses. Three-week-old hydroponically grown seedlings were stressed as described in Materials and Methods. Shoot tissues were harvested at each indicated time point. Expression level of each PgMYB gene at 0 h is set as control. The expression of *PgMYB197* gene was not detected under cold stress. The data represent the mean values of three replicates ± SD. Statistical significance of differences was tested by one-way ANOVA analysis (*p* < 0.05) and is indicated by lower case letters.

Considering the high appearance of ABREs present in the promoter region of these selected genes, we next investigated their expression pattern under ABA treatment. *PgMYB2*, *PgMYB32*, and *PgMYB159* genes were induced at 3 h after ABA application, and then, their transcript levels substantially decreased until 24 h (Figure 6). *PgMYB108* and *PgMYB197* genes were continuously upregulated by ABA and peaked at 24 h. In contrast, *PgMYB3*, *PgMYB7*, and *PgMYB37* genes were gradually downregulated along the ABA treatment. The results suggested these PgMYB genes actively respond to ABA treatment and might participate in the ABA-mediated-signaling pathway in response to environmental stresses. To test this hypothesis, we treated the three-week-old pearl millet seedlings with different abiotic stresses, such as dehydration, osmotic (i.e., PEG and NaCl), and temperature stresses, to examine their expression patterns in shoots. As shown in Figure 7, significant transcriptional changes of each candidate were observed 3 h after the onset of stress treatment. The *PgMYB3*, *PgMYB7*, and *PgMYB37* genes were all induced by dehydration, NaCl, and PEG treatments. Contrarily, The *PgMYB32*, *PgMYB79*, and *PgMYB108* genes were downregulated by the above treatment, respectively. The transcript level of the *PgMYB197* gene did not significantly change under these three treatments. Under temperature stress, five and three selected PgMYB genes responded to heat (42 °C) and cold (4 °C) stress, respectively. For instance, the *PgMYB2*, *PgMYB108*, and *PgMYB197* genes were highly induced by heat stress, while only the *PgMYB32* gene was upregulated by cold stress. Notably, the *PgMYB108* gene was responding to all stress conditions and expressed oppositely when subjected to heat and cold stress.

*Arabidopsis MYB11*, *MYB12*, and *MYB111* genes respond strongly to diverse abiotic stresses [36,37,38]; thus, we were interested in examining the transcript levels of their pearl millet homologs (i.e., *PgMYB143* and *PgMYB174*) in various tissues and under different treatments, despite their currently incomplete genome sequences. Both genes were highly expressed in shoots and exhibited similar expression pattern in response to dehydration, PEG, or NaCl treatments (Figures S6 and S7). Unlike *PgMYB143*, the transcript level of *PgMYB174* was significantly suppressed at 24 h after exposure to cold condition. Collectively, the results indicated all tested pearl millet R2R3-type MYB genes, similar to their phylogenetic homologs in *Arabidopsis*, significantly responded to distinct environmental stimulus.

## 3. Discussion

The MYB gene family, as one of the largest families of transcription factors, actively participates in the regulation of various plant biological processes [39]. To date, no comprehensive analysis of the MYB gene family has been reported in pearl millet. In the present study, a total of 208 MYB family members were identified from a pearl millet public genome database, and 193 high-confidence members were subjected to downstream analysis. Pearl millet has almost the same number of MYB genes as foxtail millet (209), supporting their close evolutionary relationship [9,27]. Based on their structural signatures, the 193 PgMYB candidates could be divided into four subgroups, among which the R2R3-type had the most members, concordant with previous reports [7,9]. Phylogenetic and motif composition analyses indicated PgMYB members of the same types usually clustered into the same clade, and there was no conserved motif that was ubiquitously presented in each PgMYB member (Figure 1 and Figure 2).

Gene duplication is a major source mechanism for new genes and can influence gene function and identity, enhancing the adaptability of related species to a variety of changing environments [28]. Both tandem and segmental duplications occurred in the MYB families [7,9]; however, only 18 pairs of segmental duplication PgMYB genes were identified (Table 1), indicating segmental duplications may be responsible for the expansion of MYB family members under purifying selection pressure. During the evolutionary process of pearl millet, there is more duplication of one gene into multiple genes between species that are more closely related (Figure 3, Tables S3–S6). Pearl millet has 40-fold more collinear MYB genes with foxtail millet than with *Arabidopsis* (Figure 3). Similarly, a genome-wide study of the PgWRKY family did not reveal any genes collinear with *Arabidopsis* [25]. This might be caused by the loss of large segments during the differentiation of dicotyledonous species. 

The R2R3-type MYB members are widely involved in response to various abiotic stress [5,39]. Genes with high homology in the same branch of the evolutionary tree usually have high sequence similarity and may also have similar functions [26,40]. According to the phylogenetic analysis of R2R3-type MYB members from Arabidopsis and pearl millet, we selected eleven PgMYB genes for tissue-specific and stress-responsive transcriptional characterization. Most of the selected PgMYB genes were more abundant in shoots than in roots (Figure 5 and Figure S6). Therefore, we focused on their aboveground transcriptional changes under different treatments. Transcriptional response of many PgMYB genes to abiotic stress exhibited similar patterns to those observed in their homologs. For instance, the *PgMYB32* gene was significantly induced by PEG or low-temperature treatment in a pattern consistent with its homologs, *AtMYB96* and *OsMYB60* (Figure 6 and Figure 7) [14,16,41]. Functional analysis revealed *AtMYB96* could integrate ABA-dependent and ABA-independent pathways to affect drought and cold tolerance in plants. The *PgMYB108* gene was strongly upregulated by the NaCl and ABA treatments and was moderately induced by dehydration, similar to its homolog in Arabidopsis and rice [42,43,44]. Considering the high abundance of ABRE *cis*-regulatory element in the promoter region and their transcriptional fluctuations during ABA treatment, we speculated that *PgMYB32* and *PgMYB108* might respectively participate in ABA-mediated-signaling pathway in response to deleterious stress. Notably, unlike *AtMYB2*, *OsMYB2* and *PgMYB108* could also respond to temperature stress (Figure 7). Similarly, the *PgMYB79* gene was strongly induced by dehydration and PEG, whereas its homolog, the *OsMYB30* gene, was an important regulator in cold tolerance [45]. Further transgenic studies are warranted to elucidate whether *PgMYB79* and *PgMYB108* are involved in the response of pearl millet to cold stress. Lately, ninety-five salt stress responsive microRNAs (miRNA) were identified from pearl millet by sequencing [46]. By searching their target database, eleven R2R3-type PgMYB genes (e.g., *PgMYB7*, *PgMYB143*, etc.) might be post-transcriptionally regulated. Although expression patterns differ from their homologs, the robust transcriptional changes to distinct abiotic stress make the PgMYB genes good candidates for genetic-engineering-mediated improvement of plant tolerance.

## 4. Materials and Methods

### 4.1. Identification and Phylogenetic Analysis of Pearl Millet MYB Genes

The whole genomic data of pearl millet were acquired from its genome-sequencing website (http://cegsb.icrisat.org/ipmgsc/, accessed on 20 August 2022). The MYB-specific conserved domain model (PF00249) [47] was downloaded from the Pfam database v35.0 (https://pfam.xfam.org, accessed on 20 August 2022) and employed this model to identify the protein sequence of pearl millet through the HMMER 3.0 program [48]. All output proteins with an e-value ≤ 1 × 10^−5^ were collected as candidate members of the pearl millet MYB family. The NCBI-CDD web server (https://www.ncbi.nlm.nih.gov/cdd/, accessed on 20 August 2022) [49] and SMART program (http://smart.embl-heidelberg.de/, accessed on 20 August 2022) [50] were used to analyze the structure of PgMYB proteins. To identify the PgMYB R2R3 members, sequence comparison was performed by the MUSCLE v5.0 [51], and the deduced amino acid sequences of the conserved structural domains of MYB were manually adjusted by MEGA11 [52]. The multiple alignment files of the R2 and R3 MYB structural domains were generated by WEBLOGO (http://weblogo.berkeley.edu/logo.cgi, accessed on 20 August 2022) with default setting. Next, physicochemical properties of PgMYB proteins were predicted by using the ExPASy (https://www.expasy.org/tools/protparam.html, accessed on 20 August 2022). A neighbor-joining (NJ) phylogenetic tree was constructed and visualized using MEGA11 and iTOL (https://itol.embl.de/, accessed on 20 August 2022), respectively [53]. Bootstrap analysis with 1000 replicates was performed to calculate the reliability of the NJ tree.

### 4.2. Subcellular Localization Analysis of Pearl Millet MYB Members

The subcellular localization analysis was conducted by using PSORT website (https://wolfpsort.hgc.jp/, accessed on 20 August 2022).

### 4.3. Synteny and Ka and Ks Analysis of PgMYB Homologous Pair

To compare protein homology among PgMYB members and those of other species, genomic information was obtained from the Ensembl Plants Database (http://plants.ensembl.org/index.html, accessed on 20 August 2022) for foxtail millet (*Setaria italica*), rice (*Oryza sativa*), *Brachypodium* (*Brachypodium distachyon*), and *Arabidopsis* (*Arabidopsis thaliana*), respectively. The covariance between PgMYB members and the other species was calculated by MCScanX [29] and was visualized by using the advanced circle function of TBtools [54]. To further estimate the replication events of the PgMYB gene, Ka (non-synonymous substitutions) and Ks (synonymous substitutions) of the cognate gene pairs were calculated using the Ka/Ks calculation function of TBtools. The predicted time of detergence (T) was calculated as described previously [55].

### 4.4. Conserved Motifs and Gene Structure Analysis

The conserved motifs of the PgMYB proteins were predicted by MEME Suite 5.4.1 webserver (http://meme-suite.org/, accessed on 20 August 2022) [56]. The maximum number of motifs found was 10, and other parameters were used as default values. The exon and intron information of PgMYB genes were extracted from pearl millet genomic information data.

### 4.5. Promoter Analysis of PgMYB Genes

The upstream genomic DNA sequences (2.0 kb) of PgMYB genes were used for promoter analysis, and stress-related *cis*-regulatory elements were predicted using the PlantCARE program (http://bioinformatics.psb.ugent.be/webtools/plantcare/html/, accessed on 20 August 2022) [57].

### 4.6. Expression Analysis of PgMYB Genes

Pearl millet seeds were surface sterilized with 5% hypochlorous acid (HClO) for 2 min, rinsed three times with double-distilled water (ddH_2_O), then sterilized twice by immersion in 70% ethanol for 2 min, and rinsed three times with ddH_2_O again. After imbibed in ddH_2_O for 10 min, the seeds were placed in Petri dishes and germinated in dark condition at 30 °C for 24 h. Consistently, the germinated seedlings were transplanted into hydroponic boxes with customized nutrient solution (91.4 mg L^−1^ NH_4_NO_3_, 32.14 mg L^−1^ NaH_2_PO_4_, 158.22 mg L^−1^ MgSO_4_, 71.4 mg L^−1^ K_2_SO_4_, 88.6 mg L^−1^ CaCl_2_, 1.5 mg L^−1^ MnCl_2_·4H_2_O, 0.074 mg L^−1^ (NH_4_)_6_Mo_7_O_24_·4H_2_O, 0.934 mg L^−1^ H_3_BO_3_, 0.035 mg L^−1^ ZnSO_4_·7H_2_O, 0.031 mg L^−1^ CuSO_4_·5H_2_O, 4.62 mg L^−1^ FeCl_3_, 11.9 mg L^−1^ Citric acid monohydrate). Seedlings were cultured in the growth chamber maintained at 28 °C/24 °C (16-h-light and 8-h-dark cycle, 120 µmol^−2^ s^−1^ light intensity). Three-week-old plantlets with uniform and robust growth were selected for abiotic stress treatments. For osmotic stress, the seedlings were placed in a nutrient solution containing 20% (*w*/*v*) PEG6000 and 150 mM NaCl, respectively. For dehydration treatment, the seedlings were placed on the Whatman 3 MM paper to dry in ambient temperature. For temperature stress, the seedlings were transferred to the growth chamber at 42 °C and 4 °C, respectively, to simulate heat and cold stresses. For ABA treatment, a solution containing 100 μM ABA (Macklin, Shanghai, China) was applied to the seedlings. The shoot tissues of pearl millet were collected at the indicated time points, frozen immediately in liquid nitrogen, and stored in a −80 °C freezer for further analysis. 

For tissue-specific-expression analysis of PgMYB genes, the root and shoot tissue of the three-week-old hydroponically grown seedlings, the leaves, stems, and spikes of soil-grown mature plants, and dry and germinating seeds were collected and stored as described above. 

Total RNA was extracted by following instructions of MolPure^®^ Plant RNA Kit (Yeasen, Shanghai, China). RNA quality was monitored by 1.0% (*w*/*v*) agarose gel electrophoresis, and the concentration were measured by using MicroDrop spectrophotometer (BIO-DL, Shanghai, China). One microgram of RNA was reverse transcribed to the first strand cDNA by PrimeScript™ reagent Kit with gDNA eraser (Takara, Kusatsu, Japan) following the manufacturer’s instructions. The reverse transcription–quantitative PCR (RT–qPCR) primers were designed using Primer Premier 5 software (PREMIER Biosoft, USA), and their sequences are listed in Supplementary Table S8. The RT-qPCR reaction was conducted on BioRad CFX96 Touch Real-Time PCR Detection System using TB Green^®^ Fast qPCR Mix (Takara, Japan). The pearl millet EF1α gene (accession: EF694165) was used as the internal control [58]. Three technical replicates were set for each sample, and the 2^-ΔΔCT^ method was used for the relative quantitation [59].

### 4.7. Statistical Analysis

Statistical analyses and plotting were performed with GraphPad Prism version 9.3 (GraphPad Software, Inc., San Diego, CA, USA). Data on transcriptional analysis were subjected to statistical analysis by one-way ANOVA.

## 5. Conclusions

In the present study, we identified 193 high-confidence pearl millet MYB genes through a genome-wide survey and performed bioinformatics analysis to uncover their physiochemical properties and phylogenetic and collinear relationships. In addition, we analyzed the transcriptional levels of eleven R2R3-type PgMYB genes in different tissues and in response to various environmental stimuli, and *PgMYB32* and *PgMYB108* genes were considered for further functional characterization. The findings of this study will facilitate us to understand the biological functions of PgMYBs in coping with climate changes and provide new candidates for subsequent generation of stress-tolerant pearl millet plants through genome editing tools.

## Figures and Tables

**Table 1:** Ka/Ks analysis and predicted divergence time of PgMYB genes.

Gene Name	Ka	Ks	Ka/Ks	T (Mya)	Duplication type	Type of Selection
PgMYB11/PgMYB155	0.05	0.35	0.14	27.2	segmental	Purify selection
PgMYB13/PgMYB158	0.25	0.49	0.52	37.8	segmental	Purify selection
PgMYB14/PgMYB161	0.41	0.97	0.43	74.8	segmental	Purify selection
PgMYB16/PgMYB166	0.23	0.40	0.57	30.7	segmental	Purify selection
PgMYB18/PgMYB169	0.22	0.89	0.24	68.2	segmental	Purify selection
PgMYB21/PgMYB189	0.22	1.17	0.19	89.8	segmental	Purify selection
PgMYB57/PgMYB73	0.13	0.50	0.26	38.4	segmental	Purify selection
PgMYB65/PgMYB129	0.20	0.73	0.27	56.0	segmental	Purify selection
PgMYB66/PgMYB133	0.26	1.38	0.19	105.8	segmental	Purify selection
PgMYB70/PgMYB100	0.21	1.38	0.15	106.2	segmental	Purify selection
PgMYB74/PgMYB99	0.33	0.61	0.55	46.6	segmental	Purify selection
PgMYB75/PgMYB97	0.41	2.13	0.19	163.8	segmental	Purify selection
PgMYB77/PgMYB92	0.25	0.40	0.62	30.7	segmental	Purify selection
PgMYB78/PgMYB93	0.23	0.51	0.46	39.1	segmental	Purify selection
PgMYB80/PgMYB90	0.17	0.67	0.25	51.5	segmental	Purify selection
PgMYB81/PgMYB87	0.17	0.43	0.39	33.2	segmental	Purify selection
PgMYB139/PgMYB193	0.16	0.47	0.35	36.3	segmental	Purify selection
PgMYB141/PgMYB191	0.27	0.45	0.59	34.9	segmental	Purify selection

## Data Availability

The data and materials that support the findings of this study are available from the corresponding authors upon reasonable request.

## Supplementary Materials

The following supporting information can be downloaded at: https://www.mdpi.com/article/10.3390/ijms24032484/s1.
