# Supplementary material for: Genome-Wide Identification and Transcriptional Analysis of the MYB Gene Family in Pearl Millet (Pennisetum glaucum)
Source: Int J Mol Sci. 2023 Jan 27;24(3):2484. doi: 10.3390/ijms24032484 (PMC9916650; doi:10.3390/ijms24032484)
Supplement: Supplementary file 1 [file ijms-24-02484-s001.zip › Supplemental figures.pdf]

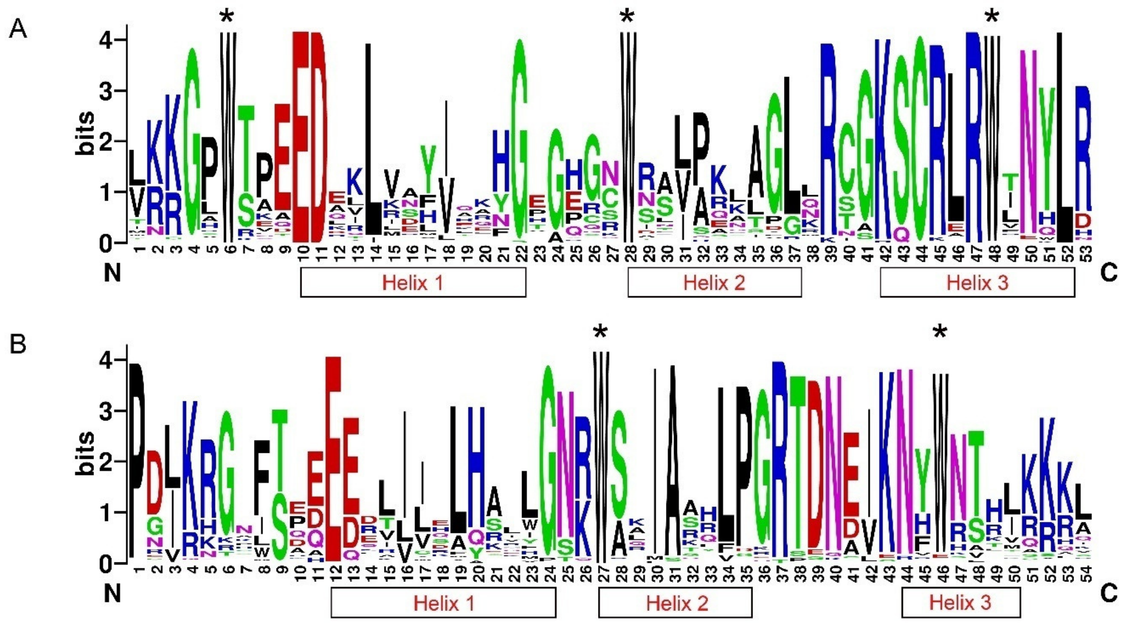

Supplemental Figure S1. Sequence logos of the R2 (A) and R3 (B) MYB repeats. The bit score exhibits the information content for each position in the sequence. The positions of the three α-helices are marked, respectively. Asterisks indicate the highly conserved tryptophan residues (W) in the MYB domain.

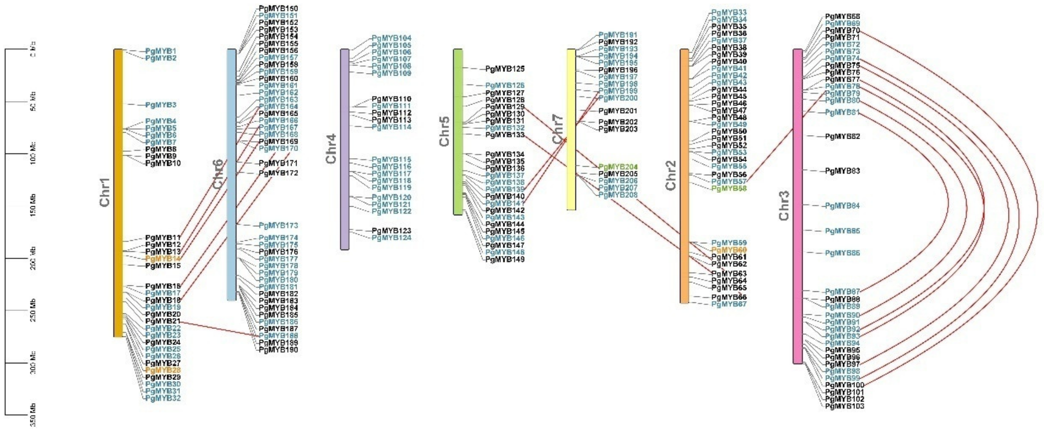

Supplemental Figure S2. Chromosomal localization of 208 PgMYB genes. Pear millet chromosomes are represented in different colors. Members belong to MYB-R2R3, MYB-related, MYB-3R, and atypical subfamilies are represented in blue, black, orange, and green font, respectively. Red lines indicate 18 duplicated PgMYB gene pairs.

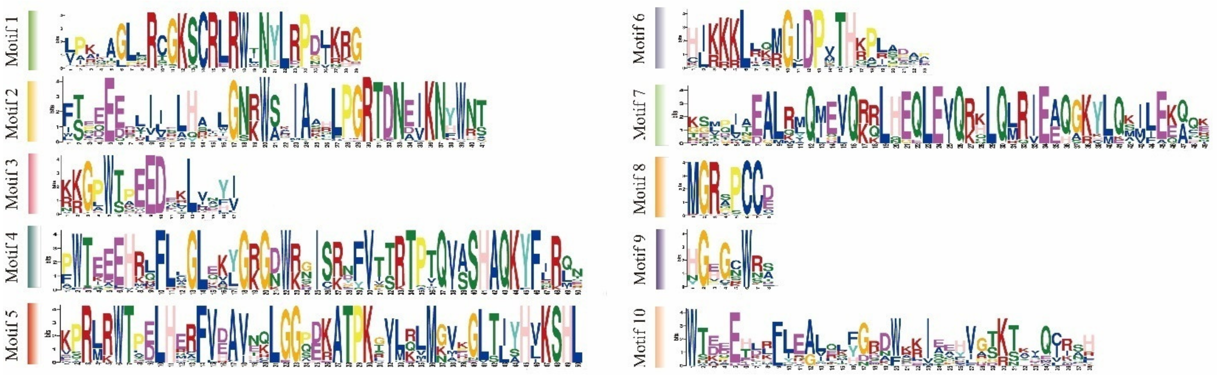

Supplemental Figure S3. Sequence logos of PgMYB conserved motifs. The color codes of each motif correspond to those outlined in Figure 1.

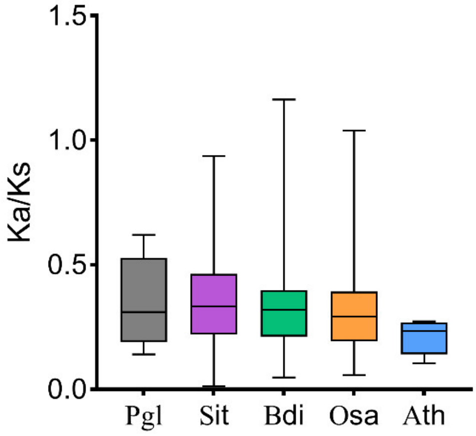

Supplemental Figure S4. Ka/Ks analysis of orthologous MYB gene pairs in pearl millet (Pgl), foxtail millet (Sit), Brachypodium (Bdi), rice (Osa), and Arabidopsis (Ath). Horizontal line of box plot is the median and whiskers display minimum and maximum values. Colors correspond to the legend provided in Figure 2.

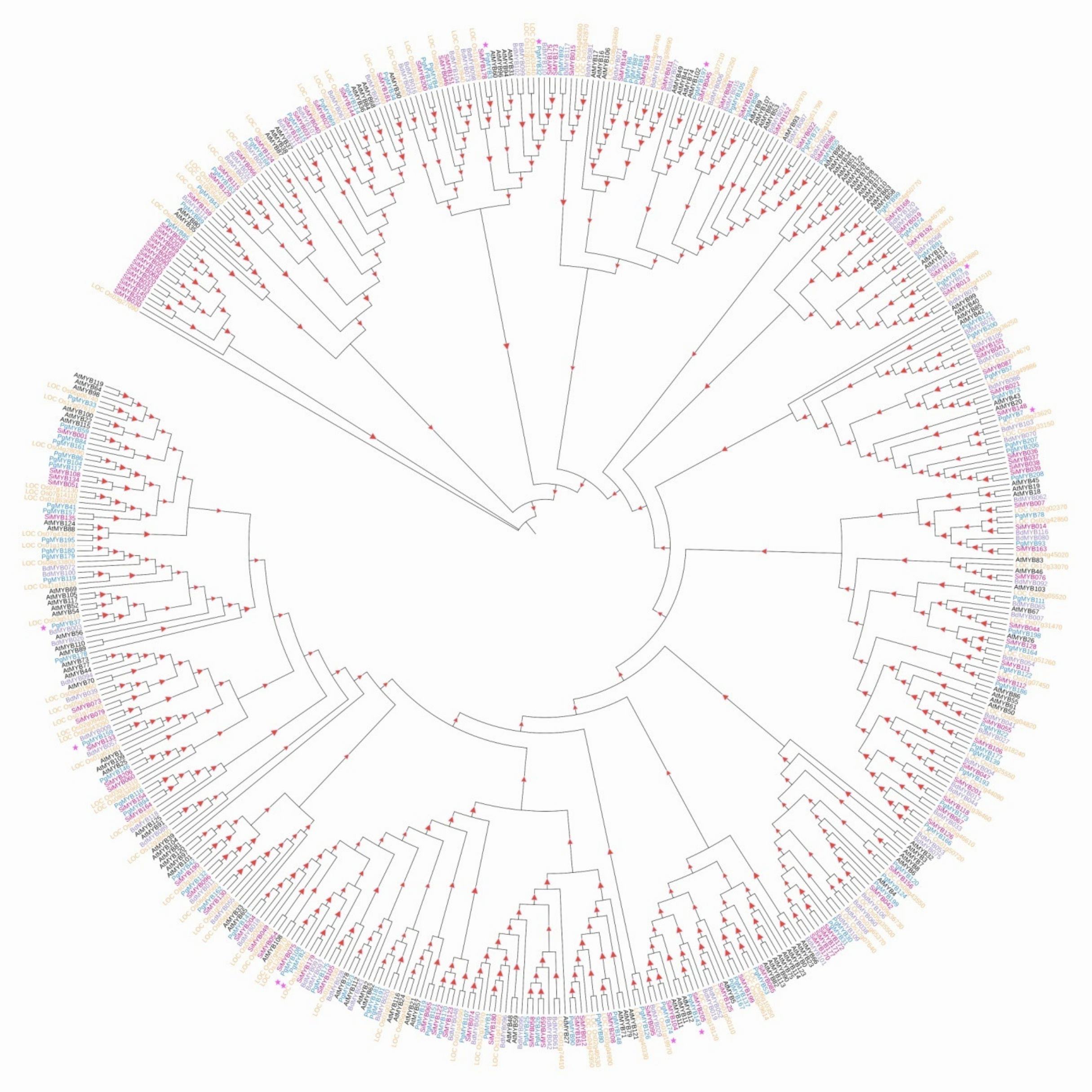

Supplemental Figure S5. Phylogenetic analysis of MYB-R2R3 members in Arabidopsis (black), Brachypodium (purple), rice (orange), foxtail millet (pink), and pearl millet (blue). Red stars indicate the eleven PgMYB genes that selected for transcriptional analysis. Red triangles represent bootstrap (1000 repetitions) and range from 0.003 to 1.

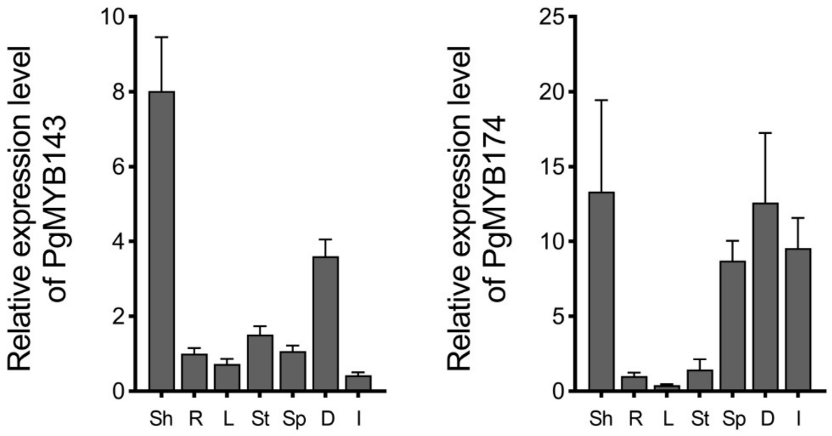

Supplemental Figure S6. Tissue specific expression pattern of *PgMYB143* and *PgMYB174* gene. Expression level of each PgMYB gene in root is set as control. Sh and R, shoot and root of three-week-old seedling; L, mature leaves; St, stems; Sp, spikes; D, dry seeds; I, geminating seeds. The data represent the mean values of three replicates ± SD.

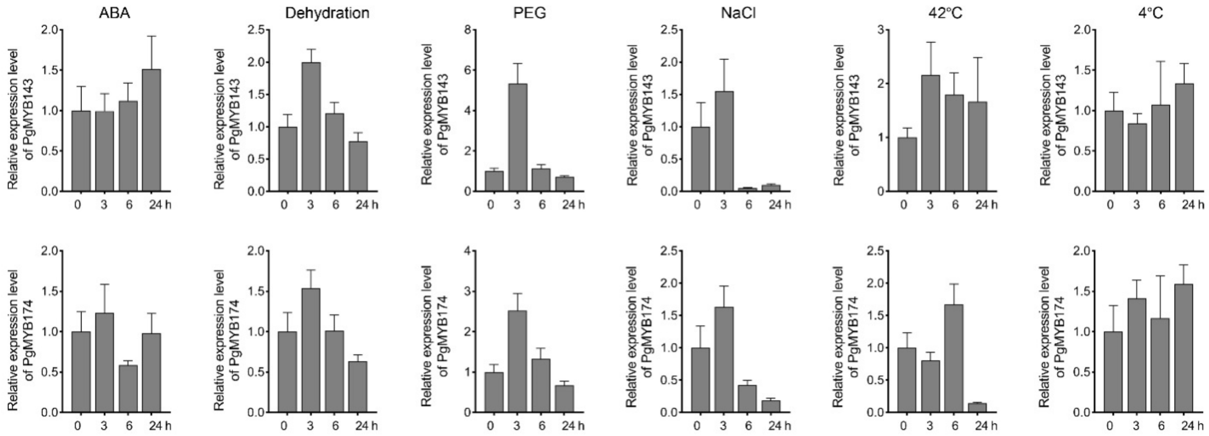

Supplemental Figure S7. Expression analysis of *PgMYB143* and *PgMYB174* gene in response to different abiotic stresses and ABA treatment. The data represent the mean values of three replicates ± SD.
